# Impacts of biodiversity-dependent ecosystem service debts on the safe operating space of social-ecological systems: a theoretical modelling study

**DOI:** 10.1098/rspb.2025.1744

**Published:** 2025-08-27

**Authors:** Maarten B. Eppinga, Nathalia Pérez-Cárdenas, Martin O. Reader, Dominic A. Martin, Maria J. Santos

**Affiliations:** ^1^ Department of Geography, University of Zurich, Zurich, Switzerland; ^2^ Department of Environmental Sciences, Wageningen University and Research, Wageningen, The Netherlands

**Keywords:** critical transitions, ecosystem services, extinction debt, natural resource management, sustainable development, systems analysis

## Abstract

The rapid environmental changes of the Anthropocene create legacy effects that may shape future Earth system responses. One significant legacy effect is the species extinction debt caused by past habitat destruction. As biodiversity underpins ecosystem services vital to human societies, social-ecological systems may, in turn, be subjected to biodiversity-dependent ecosystem service debts. While biodiversity-dependent ecosystem service debts have been quantified with analytical approaches, less attention has been paid to their potential impact on social-ecological system trajectories. We performed a theoretical study of a dynamical systems model that includes the possibility of biodiversity-dependent ecosystem service debts emerging from past habitat destruction. Our results suggest that these debts reduce systems’ safe operating spaces and create environmental tipping points associated with critical transitions in system states. These transitions, however, may include long transients of apparent stability, making it difficult to identify cause and effect. Notably, biodiversity-dependent ecosystem service debts may drive initial phases of apparent recovery after disturbance, still followed by system collapse. Our theoretical findings highlight the need to consider biodiversity-dependent ecosystem service debts for sustainable management of social-ecological systems. Furthermore, these results suggest that social-ecological systems’ safe operating spaces cannot be reliably inferred from recent observations of apparent system stability.

## 1. Introduction

The Anthropocene is characterized by distinct human-driven changes in climate, land use and biodiversity [1,2]. These environmental changes impact ecosystem functions and the provision of services on which humans depend [3,4]. An understanding of the social-ecological interdependencies and feedbacks between anthropogenic environmental changes and ecosystem responses is needed to develop future scenarios of sustainable development [5]. Assessments of ecosystem responses need to consider that current anthropogenic environmental changes are happening at an unprecedented pace [6]. Since Earth system components do not respond instantaneously to environmental changes [7], future ecosystem responses will partly depend on past dynamics [8]. Increasing evidence shows that such legacy effects may affect system stability [9,10]. Therefore, it is important to consider these effects when evaluating future trajectories and the safe operating space for social-ecological systems [11–13].

Within the ecological subsystem, one significant legacy effect is the species extinction debt [14,15]. This refers to delayed species extinctions as a consequence of ecosystem perturbation [16]. These ecosystem perturbations may result from a variety of anthropogenic drivers, including land use change, climate change, introduction of invasive alien species and shifts in management regime [16,17]. Among these drivers, land use change that destroys or degrades habitats has been identified as a major cause of species extinctions [18]. Extinction debts complicate the link between land use change and biodiversity impacts because their magnitude depends on the type of land use change, as well as the characteristics of the affected ecosystem and species pool [19].

For example, when land use change completely destroys the habitat of short-lived sessile species without nearby source populations, local species extinction may occur relatively quickly [19,20]. However, a gradual reduction in habitat (quality) accompanying land use change will likely lead to additional species loss over time, due to reduced populations or disrupted interactions [21]. Co-extinctions, the loss of species directly or indirectly from other extinctions, further contribute to the biodiversity impacts of land use change [22]. Measuring the time scales over which (co-)extinctions occur is challenging, but inferences from empirical data suggest that it can take decades to centuries to reach a new equilibrium with a reduced number of species after habitat destruction [14,15,20]. Within the social-ecological systems framework, we then conclude that land use change driven by the social subsystem can have significant impacts on the ecological subsystem, but potentially with substantial time lags [23].

Social-ecological system feedbacks can emerge when these changes in the ecological subsystem, in turn, affect the social subsystem [24,25]. There is growing evidence that biodiversity mediates the level of ecosystem functioning [26]. Specifically, biodiversity loss reduces the efficiency of ecosystem resource utilization, recycling and disease resistance [27]; directly or indirectly reducing the extent and diversity of ecosystem services provided [28]. This means that species extinction debt from past land use change will also incur an ecosystem service debt [21,23]. Analytical approaches combining insights from island biogeography and biodiversity–ecosystem functioning research have created a framework to quantify ecosystem service debt due to habitat loss [21,23]. However, integrating models that focus individually on biodiversity, ecosystem functioning or ecosystem service provisioning remains challenging [29].

An important consequence of biodiversity-dependent ecosystem service debts is that they complicate the navigation of social-ecological systems towards their safe operating spaces [30,31]. This navigation involves decisions regarding resource use [11,12,32], land use [33], ecosystem preservation and restoration [4,34] and the (monetary) valuation of ecosystem services [23]. Dynamical systems theory has shown how legacy effects, time lags between drivers and system responses, can narrow down the safe operating space [9,33]. Furthermore, for social-ecological systems on an unsustainable trajectory, the timing of navigational measures can be critical [35,36]. Therefore, understanding how biodiversity-dependent ecosystem service debts affect the navigation of safe operating spaces requires dynamical modelling [31]. These models can then be used to study how alternative management scenarios can guide the social-ecological system within safe bounds, despite external pressures and disturbances [13].

In this study, we expand a previously developed consumer-natural resource model [35,37] by integrating biodiversity-dependent ecosystem service debts. We use a minimal modelling approach (e.g. [38]), focused on gaining an understanding of the potential impact of biodiversity-dependent ecosystem service debts on system dynamics, using clear assumptions that limit the interference of other mechanisms hampering attribution of impacts [39]. Specifically, our goal is to provide a conceptual understanding of how biodiversity-dependent ecosystem service debts may affect a system’s stability and transient dynamics. Our first pair of research questions addresses system stability. (i) How does the inclusion of the biodiversity–ecosystem functioning link affect the existence and stability of system equilibrium points? (ii) How does the biodiversity–ecosystem functioning link affect a system’s safe operating space? For transient dynamics, we address two additional questions. (iii) How do biodiversity-dependent ecosystem service debts modify transient system dynamics? (iv) How do biodiversity-dependent ecosystem service debts modify the impact of abrupt disturbances on current system dynamics? Finally, we discuss how the findings of our theoretical model analysis may aid future studies focusing on the sustainable management of social-ecological systems, including further model developments that could relax some of our simplifying assumptions.

## 2. Material and methods

### (a) Model description

**Figure 1.**
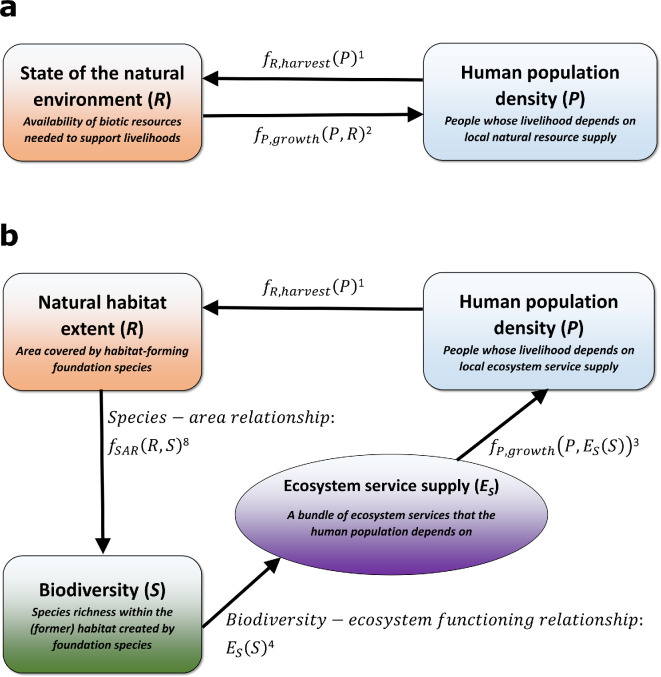
Schematic overview of the model components. (a) The baseline model does not contain biodiversity-dependent ecosystem service debt, in contrast to the extended model visualized in panel (b). Arrows point from driver to response variables. Superscripts refer to equation numbers in the main text in which the model function is quantified.

The baseline for this study was a human consumer–natural resource model system analysed in previous studies [35,37]. This model provides a stylized description of interactions between humans and natural resources, as it considers a fixed resource harvesting strategy and a uniform resource-based constraint on population growth (figure 1a). Here, we follow this baseline model to describe natural resource dynamics. Specifically, we consider natural resources as the terrestrial foundation species that create the habitat for other species. Foundation species tend to be abundant, at or near the base of directional interaction networks and highly connected to many other species through non-trophic interactions [40]. Hence, these species constrain local and regional biodiversity and strongly influence ecosystem dynamics [40]. We model the habitat that terrestrial foundation species such as trees provide to other organisms [41]. We assume that in the absence of humans, these foundation species would grow logistically, reaching a carrying capacity constrained by the system area [35]. In this study, we consider foundation species that are also an extractable resource for humans (e.g. timber production [37]). Specifically, we assume that resource harvesting will linearly increase with human population density. This assumption summarizes the expected resource harvesting dynamics for systems with open access governance regimes, in which consumers fully depend on local resource provisioning, and harvesting activities are divided in space according to an ideal free distribution [35]. Under these physical and socio-economic constraints, the natural resource dynamics are described by


(2.1) $$\frac{dR}{dt}=f_{R,growth}\left(R\right)-f_{R,harvest}\left(P\right)=c_{0}R\left(1-\frac{R}{K_{max}}\right)-hP,$$

in which the state variable R is the population size of the foundation species (units: number of resource units km^−2^), which is constrained by the system’s carrying capacity $K_{max}$ (number of resource units km^−2^). The state variable P (people km^−2^) is the human population size within the system. The parameter $c_{0}$ (year^−1^) is the maximum relative growth rate of the foundation species, whereas h (number of resource units people^−1^ year^−1^) indicates the per (human) capita utilization rate of the foundation species.

As noted above, the baseline model assumes that natural resource availability constrains human population dynamics. Previous studies have derived this dependency from the physical and socio-economic constraints already mentioned [35]. In addition, it is assumed that the amount of time and energy needed to harvest a required amount of resources increases with decreasing natural resource availability [35]. Then, due to metabolic constraints on the time and energy that can be spent on resource harvesting activities, the human population’s carrying capacity becomes proportional to the natural resource availability within the system [35,42]. It was also assumed that the human population would grow logistically towards this resource-based carrying capacity [35,37], yielding the baseline model formulation (figure 1a):


(2.2) $$\frac{dP}{dt}=f_{P,growth}\left(P,R\right)=rP\left(1-\frac{P}{qR}\right).$$

In this study, we modelled human population dynamics differently from the baseline model, assuming that they not only depend on the foundation species as an extractable resource, but on additional ecosystem services as well (figure 1b). While the human dependency on some provisioning ecosystem services may reflect the foundation species described above (e.g. consumption of species at higher trophic levels in the food web), for regulating services, the dependency would not be directly linked to energy or nutrient flows [3]. We make the simplifying assumption that there is a bundle of ecosystem services that is not substitutable and that the human population depends on. Furthermore, research has shown how ecosystem service provisioning depends on ecosystem functioning, which in turn may depend on species diversity within the system [21]. In this study, we include these dependencies in a stylized manner, assuming a direct dependency of ecosystem service supply on species diversity [28]. Hence, we can describe human population dynamics as follows (figure 1b):


(2.3) $$\frac{dP}{dt}=f_{P,growth}\left(P,E_{S}\left(S\right)\right)=rP\left(1-\frac{P}{qE_{S}\left(S\right)}\right),$$

in which r (year^−1^) is the maximum relative growth rate of the human population, $E_{S}\left(S\right)$ (number of service units km^−2^) quantifies the extent of ecosystem service supply and q (people per service unit) specifies how many humans can be sustained per unit of ecosystem service supply. As q is constant over time, impacts on the human population dynamics through changes in ecosystem service utilization, e.g. due to technological improvements [33] or behavioural changes [43], are not explicitly considered.

As shown in equation (2.3), it is assumed that there is a biodiversity–ecosystem functioning relationship in that the extent of ecosystem services provided depends on biodiversity (figure 1b). To quantify this dependency, we follow the approach of Isbell *et al*. [21], who assumed that the supply of ecosystem services increases with biodiversity. Specifically, we modelled a saturating response of ecosystem service supply to increasing biodiversity [27]:


(2.4) $$E_{S}\left(S\right)=E_{S,max}\frac{S}{c_{1}+S},$$

in which $E_{S,max}$ (number of service units km^−2^) is the maximum level of ecosystem services supplied within the system, S (number of species) is the system’s biodiversity and $c_{1}$ (number of species) is a parameter determining how quickly ecosystem service supply saturates with increasing biodiversity.

The third state variable of the model is biodiversity, S, measured by the number of species in the system (as noted above). While biodiversity can be quantified with numerous metrics, biodiversity–ecosystem functioning relationships most often use species richness [26]. We assume that species richness increases with habitat area, following previous studies quantifying the species–area relationship (e.g. [44]). This relationship implies that for a given habitat area, immigration and local extinction rates will drive the system towards an equilibrium, i.e. a constant number of species [45]. This equilibrium number of species increases with area following a saturating relationship:


(2.5) $$S_{eq}\left(A\right)=S_{max}\frac{\frac{A}{A_{max}}}{c_{2}+\frac{A}{A_{max}}}=S_{max}\frac{A}{c_{2}A_{max}+A},$$

in which $S_{eq}\left(A\right)$ (number of species) is the equilibrium level of biodiversity, $S_{max}$ (number of species) is the maximum number of species that could reside in the system, which requires that the available habitat area, A (km^2^), is at its maximum value $A_{max}$. The parameter $c_{2}$ (unitless) controls the rate at which the equilibrium number of species increases with an increasing amount of habitat.

Following the principles of island biogeography, equation (2.5) can be used to describe how habitat destruction leads to species loss [14]. On the other hand, habitat recovery can also lead to subsequent increases again [4]. From a dynamical perspective, the change in species richness over time can be described by [34]


(2.6) $$\frac{dS}{dt}=-d\left(S-S_{eq}\left(A\right)\right),$$

in which the parameter d (yr^−1^) is the system’s relaxation rate, controlling the rate at which species immigration and local extinction processes drive the system towards the equilibrium number of species. Note that equation (2.6) implies an extinction debt when $S>S_{eq}$, but also an immigration credit (e.g. [46]) when $S<S_{eq}$. The latter could occur from an increase in habitat extent due to ecosystem restoration, which may lead to an increase in successful species immigration rates at a similar time lag as for species extinctions [46]. Through equations (2.4) and (2.6), we implicitly assume that immigration credits return ecosystem functioning and service provisioning to the system to the same extent as lost through extinction. This assumes that each species contributes equally to ecosystem service supply, which is a simplification [47]. For example, non-native species may be disproportionately represented in immigration [48] and provide fewer ecosystem services than native species [49].

Equations (2.5) and (2.6) indicate that the equilibrium number of species, $S_{eq}\left(A\right)$, depends on the available habitat area. We assume that this habitat area is proportional to the population size of the habitat-forming foundation species:


(2.7) $$\frac{A}{A_{max}}=\frac{R}{K_{max}}.$$

Combining equations (2.5)–(2.7) then yields an implementation of the species–area relationship to describe biodiversity dynamics (figure 1b):


(2.8) $$\frac{dS}{dt}=f_{SAR}\left(R,S\right)=-d\left(S-\frac{S_{max}\frac{R}{K_{max}}}{c_{2}+\frac{R}{K_{max}}}\right)=d\left(\frac{S_{max}R}{c_{2}K_{max}+R}-S\right).$$

Thus, in summary, we developed a dynamical model that includes three state variables: biodiversity (measured as species richness), habitat extent (described by foundation species population density) and human population density. The dynamics of these variables are coupled (figure 1), in that: (i) the human population utilizes the foundation species, (ii) the density of the foundation species constrains system biodiversity, and (iii) system biodiversity constrains the supply of ecosystem services that the human population depends on. These dependencies are quantitatively described by equations (2.1), (2.3), (2.4) and (2.8). The interpretation of parameters and their values used in this study are shown in table 1 (see electronic supplementary material, data S1 for the motivation of these parameter values).

**Table 1.** Description of the model parameters and the system state variables. See the electronic supplementary material for further details about the information used from the references listed, and how that information is translated into the parameter values that are listed in the table.

symbol	interpretation	unit	value	references
*r*	relative population growth rate	year^−1^	0.0044	[35,37]
*q*	strength of the ecosystem service constraint on human population growth	people per service unit	1	[35,37]
$E_{S,max}$	maximum supply of ecosystem services to the human population	number of service units km^−2^	700	[35,37]
$c_{1}$	level of biodiversity at which half of the maximum ecosystem service supply is generated	number of species	100	[21]
$c_{2}$	proportion of maximum habitat area at which half the maximum level of biodiversity is maintained	unitless	0.2	[21]
$c_{0}$	productivity of the natural resource	year^−1^	0 – 0.044	[35,37,42]
$K_{max}$	carrying capacity of the local system for the natural resource	number of resource units km^−2^	700	[35,37,42]
d	relaxation rate of biodiversity to changes in habitat area	year^−1^	$\frac{\ln2}{50}$	[15,20,44]
$S_{max}$	maximum level of biodiversity	number of species	1000	[3,28,50]
h	per capita harvest rate of the natural resource (i.e. the foundation species)	number of resource units people^−1^ year^−1^	0–0.0132	[35,37]
P	human population density	people km^−2^	state variable	—
R	natural resource stock	number of resource units km^−2^	state variable	—
S	level of biodiversity	number of species	state variable	—

### (b) Analyses

Our first question addressed how the inclusion of a biodiversity–ecosystem functioning link affects the feasibility and stability of equilibria. We performed linear stability analyses and graphical analyses of the model described by equations (2.1), (2.3), (2.4) and (2.8), identifying equilibria and assessing the corresponding eigenvalues of the Jacobian matrix (e.g. [35,42]; see electronic supplementary material data S2, S3 for details). We compared these results to those obtained for the baseline model (i.e. without a biodiversity–ecosystem functioning link [35], to allow for the inference of causal relationships between modelled processes and emerging system dynamics (e.g. [51]).

Our second question focused on how the inclusion of a biodiversity–ecosystem functioning link changed the system’s safe operating space. This analysis considered two characteristics: environmental conditions (constraining productivity of the foundation species) and resource-harvesting intensity. The safe operating space can be defined as a subset of environment-harvesting combinations where a sustainable equilibrium point is feasible and stable (see electronic supplementary material, data S2 for details). We then compared the size of this emerging subset to the subset found in the baseline model [35].

The third question considered how biodiversity-dependent ecosystem service debts modified the transient system dynamics. For this analysis, we utilized results from the previous steps where bifurcation points under changing resource productivity were identified. At these bifurcation points, a further decrease in resource productivity eliminates the possibility of reaching a sustainable equilibrium point, as this point is then no longer feasible and/or stable. Instead, the formerly feasible and stable equilibrium point lingers on as a ‘ghost attractor’ (cf. [52]). Under these conditions, a system trajectory will eventually become unsustainable, but the transient dynamics towards this outcome may be influenced by the ghost attractor [52]. To examine whether such effects of ghost attractors also occurred in the extended model, we analysed the transient system trajectories for resource productivity levels below the bifurcation point, using numerical simulations.

The fourth question focused on the response to abrupt disturbances of dynamical systems subject to biodiversity-dependent ecosystem service debts. We considered a situation in which the system was on a trajectory towards a sustainable equilibrium point. From here, the system was perturbed by removing a substantial proportion of the foundation species, creating a local extinction and ecosystem service debt. The system’s trajectory after this perturbation was followed using numerical simulation.

**Figure 2.**
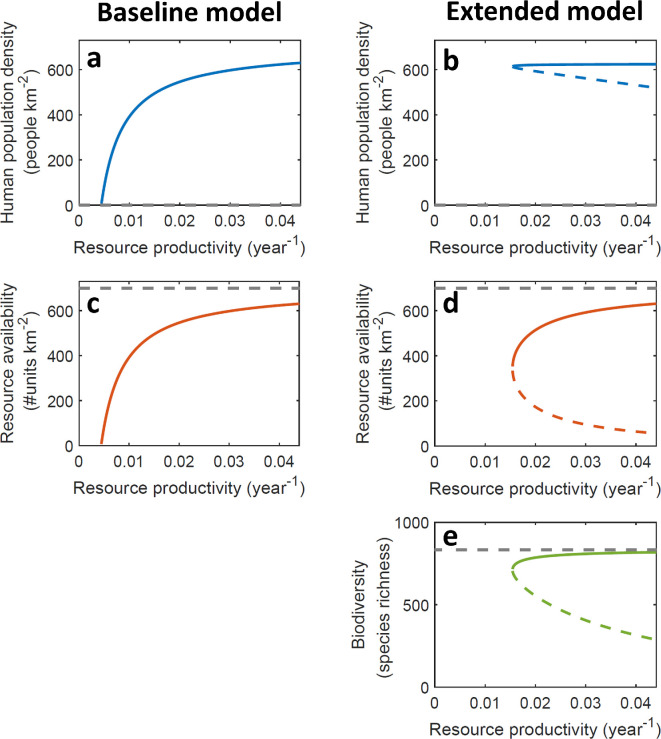
Overview of the feasibility and stability of equilibrium states of (a,c) the baseline model (describing only consumer–resource interactions) and (b,d,e) the extended model. Solid coloured lines indicate stable equilibria where all system components are present, dashed coloured lines indicate unstable equilibria and dashed grey lines indicate an unstable equilibrium point in the absence of a human population.

**Figure 3.**
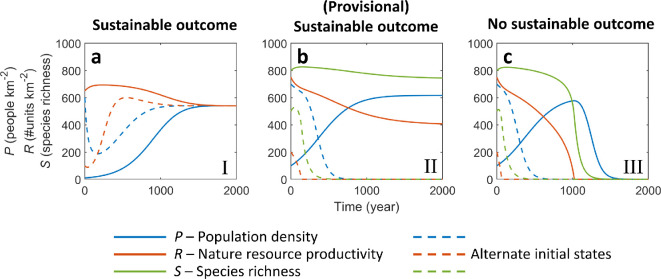
Three outcomes can be distinguished when evaluating the system’s safe operating space under constant environmental conditions. (a) A sustainable outcome that will be achieved for any initial system state. This case only occurs in the baseline model. (b) A (provisional) sustainable outcome, which the system will develop towards only from a subset of initial system states. (c) No sustainable outcome is possible, regardless of the initial system state.

**Figure 4.**
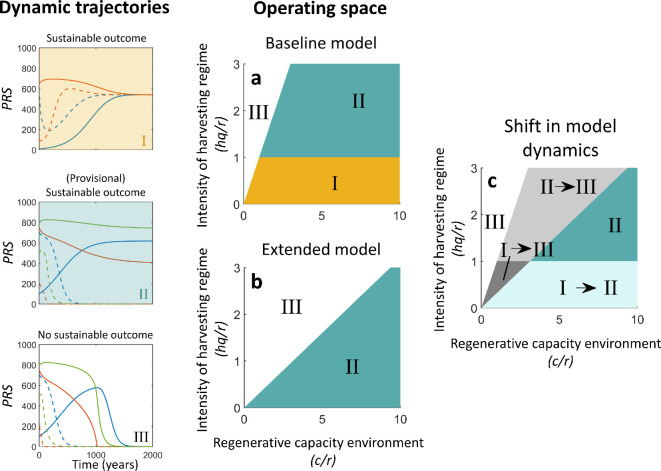
A system’s safe operating space drastically decreases due to biodiversity-dependent ecosystem service debts. (a) The safe operating space for the baseline model, as derived in a previous study [35]. (b) The safe operating space for the extended model includes a biodiversity–ecosystem functioning relationship. (c) Observed changes in dynamics between the baseline and the extended model. Grey regions indicate reductions in the safe operating space due to biodiversity-dependent ecosystem service debts. Roman numerals correspond to the dynamic trajectories shown in the inset panels.

**Figure 5.**
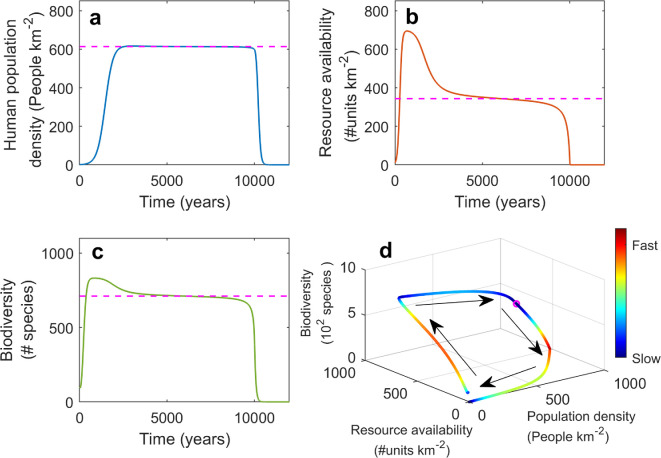
A system on an unsustainable trajectory may go through a long transient state of apparent stability, in the proximity of a ghost attractor (magenta dashed lines). During this phase, the (a) human population, (b) natural resource availability (and habitat area) and (c) biodiversity in the system may undergo little change, until a sudden system collapse occurs. (d) The long transient state of apparent stability can be explained by the presence of a ghost attractor in the state space (the magenta sphere) around which the system undergoes slow rates of change. Warmer colours indicate faster changes (calculated as $\log\left(1+\frac{\left|\Delta P\right|+\left|\Delta R\right|+\left|\Delta S\right|}{\Delta t}\right)$).

**Figure 6.**
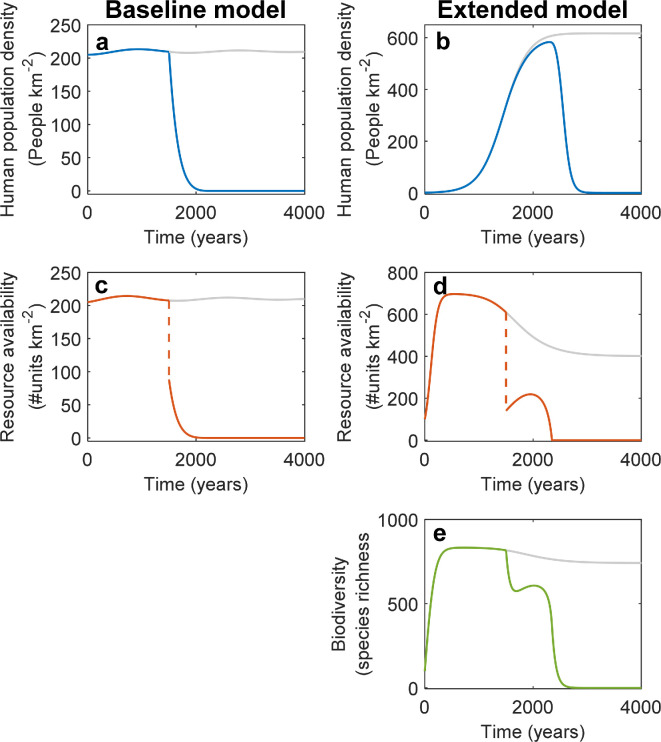
Responses to a perturbation of a system that includes biodiversity-dependent ecosystem service debts. In the baseline model, perturbations that drive system collapse create monotonic declines in (a) human population density and (c) natural resource availability. In the extended model, despite the perturbation having a small influence on (b) the human population, and even being followed by an initial phase of recovery of (d) habitat area and (e) biodiversity, the system still collapses in the long term. Grey lines show the system’s development in the absence of the perturbation.

All numerical model simulations were carried out using a fifth-order Runge–Kutta integration method implemented in MATLAB (ode45, MATLAB v. 9.0 [53]). Scripts reproducing the analyses (figures 2–6) are compatible with the open source software Octave (v. 10.2.0 [54]), and are provided as electronic supplementary material.

## 3. Results

### (a) Biodiversity–ecosystem functioning relationships create tipping points

In the absence of a biodiversity–ecosystem functioning link, the system responded in a gradual way to changes in natural resource productivity (figure 2a,c). With a biodiversity–ecosystem functioning link, however, a tipping point in natural resource productivity emerges, below which the sustainable equilibrium point of the system abruptly ceases to exist (figure 2b,d,e). Technically, this transition is described as a fold bifurcation (see electronic supplementary material, data S2 for details). When approaching this tipping point, the human population density remains relatively high compared with the baseline model (figure 2a,b). While resource availability declines substantially when approaching the tipping point (figure 2d), ecosystem service provisioning remains relatively high, as the biodiversity response is more subdued (figure 2e). This difference can be explained by the underlying species–area relationship, which displays stronger changes in biodiversity in smaller remaining areas (see electronic supplementary material, data S1 for details). We found that these results did not depend on the specific functional forms of the biodiversity–ecosystem service link (equation (2.4)) and the species–area relationship (equation (2.5)) used (see electronic supplementary material, data S4 for details). Nevertheless, for the parametrization shown (table 1), the minimum level of natural resource productivity at which the system could reach a sustainable equilibrium substantially increased when the biodiversity–ecosystem functioning link was included (figure 2). We found that this finding was robust to substantial variation in the parameters quantifying the biodiversity–ecosystem service link (equation (2.4)) and species–area relationship (equation (2.5)) and thus not dependent on the specific model parametrization used (see electronic supplementary material, data S5 for details). The increase in the required minimum level of natural resource productivity suggests that navigation within the system’s safe operating space may become more challenging, as we address in §3b.

### (b) Biodiversity–ecosystem functioning relationships reduce safe operating spaces

In the baseline model, a sustainable outcome was reached for low levels of harvesting intensity, regardless of the initial system state (figure 3a, region I in figure 4). For higher levels of harvesting, a sustainable outcome was possible for a subset of initial system states, provided that resource productivity was sufficient (figure 3b, region II in figure 4). In other cases, no sustainable outcome was possible, regardless of the initial system state (figure 3c, region III in figure 4).

Adding the biodiversity–ecosystem functioning link to the model reduced systems’ safe operating spaces in two ways (figure 4b,c). First, a sustainable outcome was possible for a narrower set of resource productivity–human harvesting intensity combinations (figure 4b,c) compared with the baseline model (figure 4a). Second, for a given level of natural resource productivity and human harvesting intensity, we found that the set of initial system states that would converge to the sustainable equilibrium point was smaller (figure 4b). More specifically, while in the baseline model, a parameter range existed where all system states converged towards the sustainable equilibrium point (figure 3a, region I in figure 4), this no longer occurred in the extended model (figure 4b). This can be explained by the emerging dynamics associated with tipping points as previously described, and the existence of an unstable equilibrium point (figure 2). For the studied parametrization, the unstable equilibrium does not disappear until extremely high levels of natural resource productivity (i.e. 160*r*). Below this extremely high level, there is an unstable equilibrium that bounds the stable equilibrium’s basin of attraction, i.e. the set of system states that can converge to the stable equilibrium point. Hence, there is always a possibility of critical transitions in system states, triggered either by gradual changes in environmental conditions or by perturbations of the current system state. In the next sections, we consider the transient dynamics of these types of transitions using numerical simulation.

### (c) Ecosystem service debts drive long transients towards system collapse

The transient system dynamics showed that a collapse was not necessarily gradual (figure 5). Instead, collapse could be preceded by a long period of apparent system stability, followed by a relatively rapid decline in human population density (figure 5a), natural resource availability (figure 5b) and biodiversity (figure 5c). These system dynamics emerged in parts of the parameter space that were close to the bifurcation point where the stable, sustainable equilibrium point ceased to exist. In this part of the parameter space, the former equilibrium point acts as a ghost attractor (cf. [52]) around which system dynamics progress very slowly, explaining the prolonged period of apparent stability (figure 5d). These dynamics show that a sudden system collapse is not necessarily linked to recent perturbation or changes in environmental conditions (figure 5).

### (d) Ecosystem service debts may reverse initial responses to system perturbations

A system on a trajectory towards a sustainable equilibrium (grey lines in figure 6) could switch to a trajectory to collapse due to an external perturbation. In low-dimensional systems (including the baseline model described above), such trajectory changes are characterized by monotonic decreases in the system’s state variables (figure 6a,c). However, in the system including biodiversity–ecosystem functioning relationships, alternative dynamics emerge (figure 6b,d,e).

Specifically, when considering a sudden reduction in the foundation species-created habitat area, there was little change in human population dynamics (figure 6b). Interestingly, the system initially recovered partly from this perturbation, in that the natural resource availability initially increased after the perturbation (figure 6d). As this change constitutes an increase in habitat area, this change also drove a recovery in biodiversity (figure 6e). However, the time lag involved in these biodiversity dynamics (i.e. the extinction debt) also delayed the impact of the perturbation on ecosystem service provisioning, and hence on human population dynamics (figure 6b,d). As a result, the human population increases too strongly, given natural resource availability in the system, eventually leading to such an imbalance that a trajectory of overexploitation and system collapse occurs (figure 6b,d,e). Excluding extinction and ecosystem service debt, the same initial system perturbation would be insufficient to alter the sustainable outcome of the system’s trajectory, as per our previous observation that biodiversity-dependent ecosystem service debts reduce a system’s safe operating space (figure 4; electronic supplementary material, data S6).

## 4. Discussion

Our dynamical model analyses show how biodiversity-dependent ecosystem service debts can complicate the navigation of systems within their safe operating space (figures 2,4,5, 4–6). Our study can be described as taking a minimal modelling approach [38], utilizing a stylized model that enables analytical analysis of the effects of biodiversity-dependent ecosystem service debts on system dynamics. The results obtained with this approach can be related to potential implications for social-ecological systems in which humans depend on local ecosystem service provisioning. In the following, we first discuss the complications implied by a reduction of the safe operating space of social-ecological systems. Second, we discuss the challenges that long transient trajectories may pose for the detection of social-ecological tipping points. However, when discussing the implications of our findings for real social-ecological system dynamics, it is important to acknowledge that the impacts of biodiversity-dependent ecosystem service debts would be potentially mediated by many social and ecological processes and feedbacks that we did not explicitly consider. While this focus on a minimal modelling approach can be conducive to generating conceptual insights, it also constrains the direct applicability of these insights to the sustainable management of a specific social-ecological system of interest [51]. Hence, we also reflect on the simplifying assumptions made in the current model study and discuss important processes and feedbacks that would require consideration when working towards this latter goal. This provides a perspective for future empirical and theoretical research on social-ecological systems.

### (a) Biodiversity-dependent ecosystem service debts reduce the safe operating space

We found that biodiversity-dependent ecosystem service debts reduced the system’s safe operating space (figures 2 and 4). This is consistent with previous studies of complex systems, showing that time lags between key processes can impact the stability of equilibrium points, or their basins of attraction [9,33]. This suggests that approaches defining safe operating spaces for social-ecological systems need to consider extinction and ecosystem service debts, and the specific time scales involved. Previous estimates have suggested half-lives of 25–50 years for species extinction processes, implying that 99% of an extinction debt will be paid off after approximately 332 years, which is reasonable [15,44]. However, previous studies also suggest that this period may range between 5 and 570 years, or even up to 1000 years [16,20]. Long-lived species are particularly susceptible to extinction debts due to their slower response to habitat changes, shown by disturbance legacies in forests persisting for centuries [8]. However, successful restoration efforts can avoid paying extinction debts and limit responses to past habitat reduction [4]. As this potential increases with the characteristic half-lives of species extinction processes, the temporal window of opportunity for successful navigation towards the safe operation space may increase as well [35]. In turn, this increases the context dependency of the constraints posed by biodiversity-dependent ecosystem service debts on the successful navigation of social-ecological systems towards (narrower) safe operating spaces.

Our findings corroborate previous notions of declining biodiversity reducing the safe operating space of social-ecological systems [55,56]. These previous notions, however, emphasized how declining biodiversity reduces the resilience of systems to other stressors, such as climate change [55,56]. In contrast, our results suggest that even a delayed biodiversity response in itself comprises a mechanism that reduces the safe operating space (figure 4). Specifically, a delayed biodiversity response also constitutes a delayed response in ecosystem service provisioning [21], which may temporally extend anthropogenic overexploitation of natural resources. In the long term, this phenomenon may increase the likelihood of social-ecological system trajectories describing overshoot and collapse dynamics [33,35]. These notions highlight the need to incorporate biodiversity dynamics in the assessment of social-ecological system responses to projected environmental changes [57].

### (b) Beyond the tipping point: biodiversity-dependent ecosystem service debts driving long transient responses

When explicitly considering the biodiversity–ecosystem functioning relationship, we found that the resulting human–environment interactions created tipping points. Passing a tipping point has been linked to abrupt and rapid changes in system states [58,59]. In addition, the reduced resilience of systems when approaching a tipping point implies that progressively smaller perturbations become sufficient to trigger a critical transition between system states [6,58]. Hence, to explain observed transitions in social-ecological systems, it appears intuitive to identify recent changes or perturbations that occurred around the transition point (e.g. [60]). However, our simulations show how rapid system collapse can also occur without recent perturbations (figure 5). Specifically, we showed how systems that have passed a tipping point may still be influenced by a ‘ghost attractor’ (cf. [52]), i.e. the formerly stable equilibrium point. System trajectories that approach a ghost attractor still exhibit long periods of apparent stability, before a rapid collapse of the system occurs (figure 5).

Long transients of apparent stability due to a ghost attractor only occurred in the extended model analysed here, and thus emerge specifically due to biodiversity-dependent ecosystem service debts in our model framework. These long transient dynamics towards collapse are relevant to consider when defining safe operating spaces for social-ecological systems [7,12,55]. Interestingly, different viewpoints have emerged in this context. When a system’s equilibrium points and their stability are not known, the possibility of long transients cautions against setting safe operating space boundaries based on recent observations, as prolonged periods of apparent system stability do not necessarily reflect a sustainable trajectory (figure 5) [36,61]. Indeed, within the context of biodiversity-dependent ecosystem service debts, there is a growing recognition of the need to consider ecosystem integrity and potential lags between changes in drivers and their impacts on ecosystem service provisioning [62,63]. On the other hand, it has also been suggested that long transients allow for temporarily overshooting driver pressures, with a return to the equilibrium state being possible if pressures are reduced again over time scales shorter than the system’s transient dynamics [64]. While stylized models as presented in this study can provide a conceptual understanding of how to differentiate between these cases, more detailed model frameworks, describing specific systems with a high degree of fidelity [51], would be needed to relate empirically observed dynamics to likely attractors and losses thereof [65]. In §4c, we discuss some important directions for future developments of the model framework considered in this study.

### (c) Model limitations and perspectives for future research

The model we present belongs to a class of relatively simple models using ecological predator–prey models as a starting point to describe human–environment interactions [35,37,42]. Simple models allow for analytical analyses that enable rather complete explorations of emergent dynamics [11,66]. While such explorations can generate insights and hypotheses, it is important to acknowledge the specific assumptions of the model frameworks from which these findings emerge [24,31]. For our current study, the simplifying assumptions made impose several constraints on the extrapolation of our findings to the governance of social-ecological systems towards safe operating spaces. First, the modelled dependency of the human population on local provisioning of non-substitutable ecosystem services excludes the possibility of imports from elsewhere. In real social-ecological systems, imports may become increasingly important with an increasing population size and capital resources [42]. On the one hand, such processes may allow for system persistence under lower levels of local resource availability [42]. On the other hand, increasing dependencies on the exchange of resources with other systems may also lead to increased vulnerability and the possibility of cascading collapses of multiple local systems [67]. Hence, for the study of specific social-ecological systems, consideration of the import and export of resources across the system boundary and the processes mediating the extent of these flows is important.

Second, our modelling of resource harvesting as a process that increases linearly with human population density was based on the consideration of a system with an open access governance regime and a fixed efficiency of resource harvesting. In real social-ecological systems, these simplifying assumptions may not hold when there is adaptive governance of natural resource use ([68], which could also include changes in taxing schemes [23]. In addition, human resource demands may also change over time due to demographic transitions [66]. Importantly, these social processes may depend on the current state of the natural environment and ecosystem service provisioning. Similarly, changing efficiencies may be driven by technological advancements, where the rate of technological advance in turn may depend on the current system state [32,33]. Hence, further research could expand the current model framework by incorporating these processes. We could then assess to what extent specific governance strategies may avert the impacts of biodiversity-dependent ecosystem service debts as identified in the current study.

Third, describing changes in state variables through differential equations, as part of our minimal modelling approach, implicitly assumes mass action dynamics for the individual units described by the state variables. For the human population in particular, explicit consideration of individual behaviour, and changes therein due to changing social or ecological conditions, could identify potential alternative mechanisms of system adaptation and transformation, complementing system-level measures as described above [25]. Incorporating individual human behaviour and decision-making processes would require the use of agent-based modelling frameworks [43], but the comparison of emergent dynamics with minimal models as presented here provides a useful aid to the interpretation of results generated with agent-based models [38,51].

In summary, to assess the extent to which the potential impacts of biodiversity-dependent ecosystem service debts manifest themselves in specific social-ecological systems, extended model frameworks would be needed. While such extensions of the current model framework may limit the possibilities for analytical analyses, the question of how the observed dynamics change when additional processes and feedbacks are included is much simpler to address through numerical simulation. Developing model frameworks in such a step-wise manner is a promising approach to gain insights into increasingly complex social-ecological systems [51].

## 5. Conclusion

A key challenge of the Anthropocene is to understand how ecosystems respond to environmental changes of unprecedented rates and magnitudes. Dynamical systems theory suggests that time lags between social-ecological processes can play a crucial role in the emergent system responses. The species extinction debt is a prominent example of such a time lag. Moreover, the biodiversity–ecosystem functioning relationship implies that the extinction debt also leads to a biodiversity-dependent ecosystem service debt. Our study reveals the different ways in which ecosystem service debts from past human activities could complicate the safe navigation of systems. These findings suggest caution when defining operating space boundaries based on recent observations of system dynamics, or evaluating the impact of recent interventions based on short-term (recovery) trajectories.

## Data Availability

Octave/Matlab scripts that reproduce the analyses presented in this work (figures 2–6) have been uploaded as part of the supplementary material [69].
